# Prognostic Value of a Pyroptosis-Related Long Noncoding RNA Signature Associated with Osteosarcoma Microenvironment

**DOI:** 10.1155/2021/2182761

**Published:** 2021-11-11

**Authors:** Xinxin Bu, Jiuxiang Liu, Rong Ding, Zhi Li

**Affiliations:** ^1^Department of Pediatrics, The First Affiliated Hospital of Nanjing Medical University, Nanjing 210029, China; ^2^Department of Orthopedic Surgery, The First Affiliated Hospital of Nanjing Medical University, Nanjing 210029, China; ^3^Department of Orthopedic Surgery, Geriatric Hospital of Nanjing Medical University, Nanjing 210024, China

## Abstract

**Background:**

Osteosarcoma is the most prevalent bone cancer that affects young adults and adolescents. It is the most frequent malignancy of the bone. In spite of the fact that complete surgical resection and chemotherapy have increased the overall survival of osteosarcoma patients considerably, the prognosis remains dismal in patients with recurring and/or metastasized osteosarcoma. Thus, finding predictive biomarkers representing osteosarcoma's biological variability may result in more effective treatment for osteosarcoma patients.

**Methods:**

In this research, RNA data and clinical information were obtained from TARGET database. The risk score was calculated using a technique that incorporated both univariate and multivariate Cox regression. A variety of statistical methods were employed to assess the risk score's accuracy. These included ROC curves, nomograms, and Kaplan-Meier curves. Following that, bioinformatics studies were carried out in order to investigate the possible biological processes that influence the prognosis of osteosarcoma patients. GSEA was used to investigate the variations in pathway enrichment among the different groups of genes. To examine the disparities in the immune microenvironment, the analytical methods CIBERSORT and ssGSEA were employed.

**Results:**

We discovered three differentially expressed lncRNAs (RPARP-AS1, AC009159.3, and AC124312.3) that are linked to osteosarcoma prognosis. Kaplan-Meier analysis showed the presence of a signature of high-risk lncRNAs linked with a poor prognosis for osteosarcoma. Furthermore, the AUC of the lncRNAs signature was 0.773, indicating that they are useful in predicting osteosarcoma prognosis in certain cases. In predicting osteosarcoma prognosis, our risk assessment approach outperformed conventional clinicopathological characteristics. In the high-risk group of people, GSEA showed the presence of tumor-related pathways as well as immune-related pathways. Furthermore, TARGET revealed that immune-related functions such as checkpoint, T-cell coinhibition, and costimulation were significantly different between the high-risk and low-risk groups. LAIR1, LAG3, CD44, and CD22, as well as other immune checkpoints, were shown to be expressed differentially across the two risk groups.

**Conclusion:**

This study established that pyroptosis-derived lncRNAs had a significant predictive value for osteosarcoma patients' survival, indicating that they may be a viable target for future therapy.

## 1. Introduction

Osteosarcoma is a bone cancer that most often affects teenagers and young adults. One-fifth of the patients had metastases, with micrometastases probably appearing in the majority of the other patients, and chemotherapy and surgical excision are the standard treatments [1, 2]. Despite the fact that chemotherapy with cisplatin, doxorubicin, and methotrexate slightly increased the survival rate when compared to surgery alone, metastatic osteosarcoma was frequently recurrent and had a dismal prognosis, with only 30% of patients surviving for more than a year after diagnosis [3, 4]. In response to this unmet need, we have concentrated our efforts on the discovery of new prognostic targets for osteosarcoma.

A kind of RNA that has a length of more than 200 nucleotides and comes from the genome's noncoding region is known as long noncoding RNA (lncRNA) [5, 6]. At the transcription and posttranscriptional phases, lncRNAs regulate gene expression and perform a range of biological functions [7, 8]. Numerous studies have shown that long noncoding RNAs have a role in the development of osteosarcoma [9, 10].

Pyroptosis is a kind of programmed cell death that is linked with the production of proinflammatory mediators and cell disintegration [11, 12]. Pyroptosis causes inflammatory alterations in normal cells that promote carcinogenesis and create tumor microenvironments that are favorable for tumor growth [13, 14]. By stimulating the ERK 1/2 pathway, HMGB1, which is generated by pyroptotic epithelial cells, has been shown to accelerate the development of colorectal cancer in patients with colitis [15]. According to recent research, the induction of pyroptosis in the hypoxic core of the tumor has been shown to accelerate tumor growth and is linked with shorter overall survival [13, 16]. The ability to predict the outcome of malignancies has been shown in a number of studies, and more studies into the mechanism of pyroptosis may provide new therapeutic targets to guide future research.

Several studies have demonstrated that pyroptosis-derived lncRNAs influence solid tumor cells (such as cervical cancer, digestive cancers, and breast cancer) [17–19]. The function of lncRNAs derived from pyroptosis in osteosarcoma remains unknown. In this research, we hypothesize that many long noncoding RNAs are associated with pyroptosis genes and may help predict the prognosis of osteosarcoma patients. We can more effectively show the heterogeneity of osteosarcoma by integrating clinicopathological kinds and tumor molecular features, and we can provide a theoretical basis for osteosarcoma clinical diagnosis and prognosis. In order to accurately assess patients' prognosis, we developed a new scoring system based on three lncRNAs linked with pyroptosis.

## 2. Materials and Methods

### 2.1. Data Collection

The Xena website at the University of California, Santa Cruz (UCSC), was used to obtain high-throughput RNA-seq data and clinical characteristics from the TARGET database for 88 patients with osteosarcoma and 396 normal skeletal muscle samples from the GTEx project. The FPKM normalized estimate and log2-based transformation were used to quantify the gene expression patterns. Due to the scarcity of data on normal skeletal muscle tissue in the TARGET cohort, we used GTEx data to detect DEGs between normal and malignant tissues. Before comparing the two datasets, the expression data was standardized to FPKM values.

### 2.2. Differentially Expressed Genes in the TARGET Database

Thirty-three genes linked to pyroptosis have been obtained in the literature published (Table S1). DEGs were identified using the R software package “limma” with an absolute value of |log_2_FC| > 0.5 and an adj. *p* value <0.05. Following that, heatmaps were utilized to demonstrate the differences in pyroptosis-related gene expression between osteosarcoma and normal skeletal muscle tissues.

### 2.3. PPI Network

We used the Search Tool for Interaction Genes (STRING) database to create a PPI network in order to examine the differentially expressed 20 pyroptosis-related genes. A minimum gene interaction score of 0.7 was used as a criterion for genes in the center of the PPI network.

### 2.4. Identification of Pyroptosis-Associated lncRNAs and Development of a Prognostic Model

A Pearson correlation analysis was conducted in order to identify lncRNAs related to pyroptosis. The association between lncRNAs and pyroptosis genes was determined using their expression values. |*R*_2_| > 0.6 and *p* < 0.001 were our selection criteria. We used univariate Cox regression and multivariate Cox regression to generate the pyroptosis-related lncRNA signature. Using that data, we found three lncRNAs that are involved in pyroptosis to be potential signatures for the prognostic signature model. The model for predicting pyroptosis-associated lncRNAs was constructed utilizing three lncRNAs linked to pyroptosis that were derived using a previously established formula:

(1) $$\begin{array}{c} \text{risk score}=\sum_{i=1}^{n}expi*\beta i. \end{array}$$

In the formula, exp (*i*) and *β* (*i*) indicate the expression value and estimated regression coefficient of each pyroptosis-related lncRNA, respectively.

### 2.5. Assessment and Verification of Accuracy of Predictive Signatures

To demonstrate the connection between high-risk and low-risk populations, we utilized Kaplan–Meier survival curves. After analyzing the expression data and plotting the results, we utilized heatmaps and scatter plots to illustrate gene expression and prognosis in various groups. ROC curves were used to evaluate the predictive accuracy of the forecasts. To verify the connection between the risk score and clinicopathological features of patients, a correlation study was conducted. We conducted univariate and multivariate Cox regression studies to validate our prediction model.

### 2.6. Developing a Predictive Nomogram

A nomogram was developed to provide a reliable predictive tool for patients with osteosarcoma at 1, 3, and 5 years based on risk values and other clinicopathological characteristics. Calibration curves were then used to assess the degree of agreement between the expected and observed patient populations.

### 2.7. GSEA Analysis

The gene expression features of three pyroptosis-related lncRNAs as well as the genetic characteristics of the individuals classified into the high- and low-risk groups were evaluated in the TARGET cohort by using GSEA. The false discovery rate (FDR) was <0.25, and the *p* value was <0.05.

### 2.8. Immune Cell Characteristics Examination

The CIBERSORT website provides an annotation of the gene signature matrix (LM22) and defines 22 immune cell subtypes as listed in an annotation. To improve the accuracy of the deconvolution technique for each file, the CIBERSORT *p*-value and root mean square error were calculated using 100 permutations of the standard signature matrix. The CIBERSORT *p*-value < 0.05 was used to select and further process the osteosarcoma tissue data. The immune cell composition of the sample of TARGET cohort osteosarcoma has been examined using the CIBERSORT process.

### 2.9. Statistical Analysis

R version 4.0.2 and different R packages were used for statistical analysis, with a 2-tailed *p*-value of 0.05 signifying statistical significance. The univariate and multivariate Cox regression analyses were carried out using the “survival” package, respectively. The LASSO Cox regression analysis was performed using the “glmnet” package, and 10-time cross-validation was used to determine the best penalty parameter lambda. The “survival” package was used for Kaplan-Meier analysis and survival curves. The nomogram and calibration curve were produced using the “rms” program. The “timeROC” program conducted time-dependent ROC curve studies. On the basis of FDR, the Benjamini-Hochberg technique was employed to discover differentially expressed lncRNAs. Using “GSVA,” the ssGSEA-normalized osteosarcoma DEGs were compared to a genome (R-package).

## 3. Results

### 3.1. Pyroptosis-Related Genes Are Expressed Differently in Osteosarcoma and Normal Muscle Tissues

**Figure 1:**
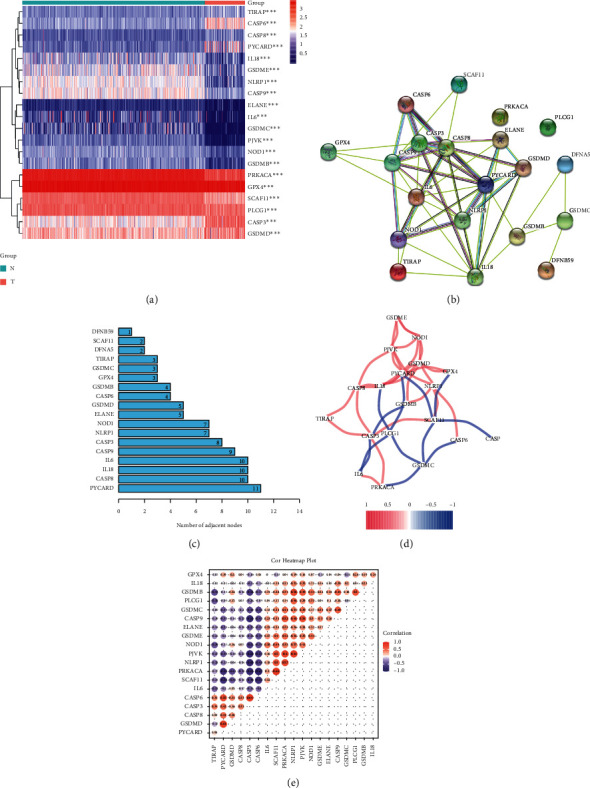
Identification of osteosarcoma and normal tissues with differently associated pyroptosis genes. (a) Heatmap graphically illustrating the differences between the two groups in the expression of genes associated with pyroptosis. (b, c) Network of protein-protein interactions (PPI) demonstrating interaction between genes expressed differently among genes linked to pyroptosis. (d) The correlation network of genes associated with pyroptosis is expressed differently. The correlation coefficients are shown in various colors. (e) Pearson's study of pyroptosis-related differential expression.

To discover the differentially expressed genes and associated activities of pyroptosis-related genes in osteosarcoma, we utilized the TARGET database to examine 88 osteosarcoma patients and 396 normal muscle tissues from the GTEx database.  Table S2 contains the full clinical features of the individuals in the TARGET database. The expression of pyroptosis-related genes differed substantially between osteosarcoma and normal muscle tissues, according to our findings (Figure 1(a)). The connection between genes linked to pyroptosis was next sought to be clarified. We constructed a PPI network comprising 20 pyroptosis-related genes using the STRING database (Figure 1(b)), and the number of nodes is displayed in Figure 1(c).  Figure 1(d) depicts the connection between these genes. PRCARD was identified as the central gene in the network, and it was discovered to interact with a total of 11 additional genes. When the connection with other genes was moderated, we discovered that PRCARD and SCAF11 had the greatest link (Figure 1(e)). The aforementioned findings showed that pyroptosis-related genes interacted with one another in osteosarcoma.

### 3.2. Enrichment Analysis of Genes Associated with Pyroptosis

**Figure 2:**
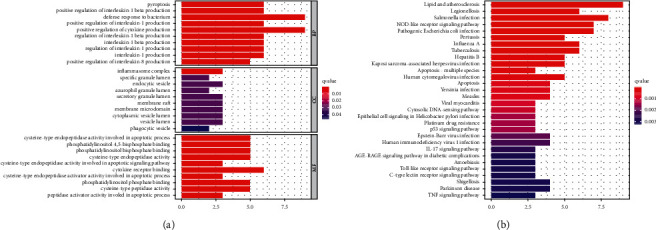
GO and KEGG analysis of genes involved in pyroptosis. (a) GO. (b) KEGG.

We discovered 20 DEGs associated with pyroptosis (14 downregulated and 6 upregulated; Table S3). BP participated in pyroptosis, positive regulation of IL-1*β* production, and defense response to bacterium. MF mainly participated in cysteine-type endopeptidase activity involved in apoptotic process, phosphatidylinositol bisphosphate binding. CC were mainly involved in inflammasome complex, specific granule lumen, and endocytic vesicle. KEGG-based analysis revealed that the DEGs were mainly involved in lipid and atherosclerosis, legionellosis, salmonella infection, and NOD-like receptor signaling pathway (Figure 2).

### 3.3. Identification of Prognostic Pyroptosis-Related lncRNAs

**Figure 3:**
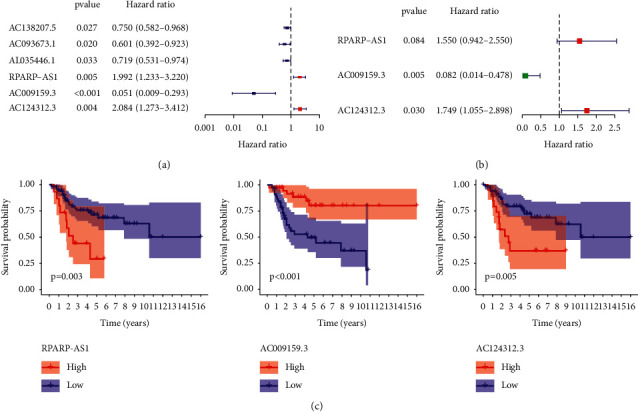
Construction of pyroptosis-related lncRNAs signature by means of univariate and multivariate cox analysis. (a) Univariate cox analysis. (b) Multivariate cox analysis. (c) Overall survival analysis of the three pyroptosis-related lncRNAs between high- and low-expression groups.

Using Pearson correlation analysis to assess correlations between the lncRNAs and the pyroptosis-associated genes, we identified 329 lncRNAs linked to pyroptosis with selection criterion of |*R*_2_| >0.6 and *p* < 0.001 (Table S4). Cytoscape was utilized to decipher the network of lncRNA-mRNA coexpression in osteosarcoma (Figure S1). In combination with clinical survival data, the results of the univariate Cox regression analysis showed a statistically significant relationship between the expression of six lncRNAs and the survival time of osteosarcoma patients (*p* < 0.001; Figure 3(a)). Three well-characterized lncRNAs (RPARP-AS1, AC009159.3, and AC124312.3) were filtered using multivariate Cox regression to create the predictive signature (RPARP-AS1, AC009159.3, and AC124312.3) (Figure 3(b)). Among the three lncRNAs, RPARP-AS1 and AC124312.3 had HR > 1, whereas AC009159.3 had HR1. RPARP-AS1 and AC124312.3 were both shown to be correlated with decreased survival and AC009159.3 was linked with improved survival (Figure 3(c)).

### 3.4. Verifying the Accuracy of Three lncRNAs Associated with Pyroptosis as a Prognostic Marker

**Figure 4:**
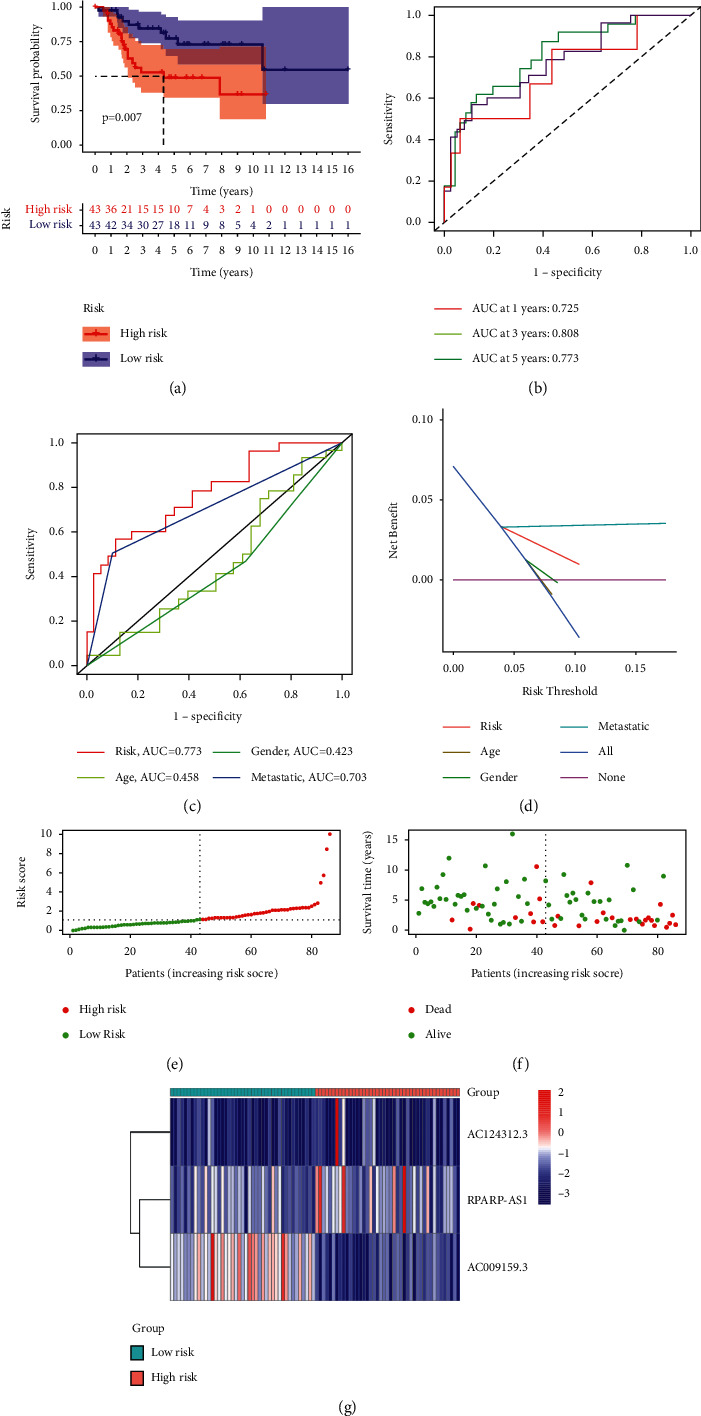
TARGET-based signature for lncRNAs associated with pyroptosis. (a) The outcome of the Kaplan-Meier curves. (b) The AUC values for the prediction of osteosarcoma survival rates at 1, 3, and 5 years. (c) The AUC values of the risk variables. (d) The risk factors' DCA. (e) Distribution of patient risk ratings. (f) Plot of risk survival status. (g) Heatmap of lncRNAs associated with pyroptosis in high- and low-risk groups.

Patients with osteosarcoma were classified into two groups based on a median cut-off value: high-risk and low-risk groups. Patients in the low-risk group had a substantially longer life and better prognosis than those in the high-risk group, according to a study of Kaplan-Meier survival curves (Figure 4(a)). With all AUC values greater than 0.7 (Figure 4(b)), the ROC curve shows that the use of risk scores to predict the prognosis of osteosarcoma patients at 1, 3, and 5 years is reliable. Furthermore, the signature's 5-year AUC was 0.773, demonstrating superior performance to traditional clinicopathological features in predicting the prognosis of osteosarcoma patients (Figures 4(c) and 4(d)). Patients with osteosarcoma were then categorized based on risk ratings derived from the prognostic profile of pyroptosis-related lncRNAs (Figure 4(e)). The scatter plot shows the association between survival and risk score in osteosarcoma patients, with those with higher risk scores having shorter survival times (Figure 4(f)). In the low-risk group, lncRNAs linked with prognostic markers were expressed differently than in the high-risk group, according to the heatmap. Risk factors were higher in high-risk patients (RPARP-AS1 and AC124312.3), whereas protective variables (AC009159.3) were higher in low-risk patients (Figure 4(g)).

### 3.5. Examining the Potential of Predicting the Risk Signature of Pyroptosis-Related lncRNAs Independently

**Figure 5:**
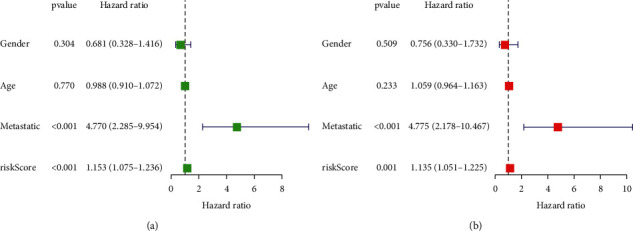
Univariate and multivariate Cox analysis for pyroptosis-related lncRNAs. (a) Univariate analysis. (b) Multivariate analysis.

The pyroptosis-related lncRNAs prognostic signature was then tested using univariate and multivariate Cox regression analysis to see whether it was an independent predictor of osteosarcoma in patients. With the exception of gender (*p* = 0.304) and age (*p* = 0.770), univariate analysis revealed that metastasis (*p* < 0.001) and pyroptosis-related lncRNAs prognostic risk score (*p* < 0.001) were significantly associated with survival (Figure 5(a)). In multivariate analysis, both pyroptosis-related lncRNAs prognostic risk score (*p* = 0.001) and metastasis (*p* < 0.001) were significantly associated with survival (Figure 5(b)). All of these statistical data showed that the risk score for pyroptosis-related lncRNAs independently predicts prognosis in patients with osteosarcoma.

### 3.6. Nomogram-Based Quantification of Clinical Parameters and Assessment of Risk Score Prognostic Accuracy

**Figure 6:**
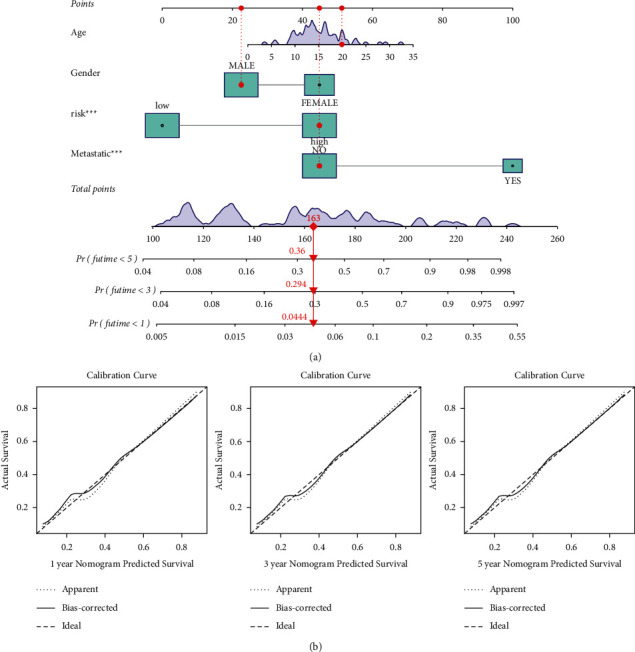
A nomogram for both clinic-pathological variables and predictive pyroptosis-related lncRNAs. (a) Predictive nomogram. (b) Calibration curve for the 1-, 3-, and 5-year predicted survival nomogram.

As part of this section, we used the nomogram based on clinicopathological characteristics as well as the prognostic signature of the pyroptotic-related lncRNAs to generate a score that could be used to evaluate the accuracy of the prediction model. For osteosarcoma patients, we developed a nomogram (Figure 6(a)) that combined multiple clinicopathological characteristics such as age, gender, histological type, grade, and the risk score associated with pyroptosis-related lncRNAs in order to accurately predict their 1-, 3-, and 5-year survival times. In the calibration study, the predicted and observed OS at 1, 3, and 5 years for osteosarcoma patients were in good agreement (Figure 6(b)).

### 3.7. Gene Set Enrichment Analyses

**Figure 7:**
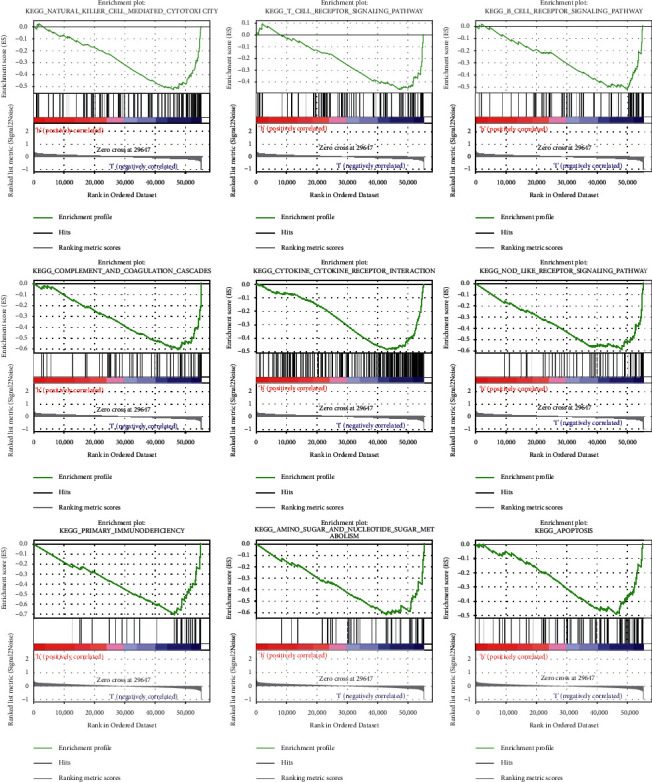
GSEA analysis for pyroptosis-related lncRNAs based on TARGET.

We performed GSEA comparing the high-risk and low-risk groups to explain how the pyroptosis-related signature lncRNAs involving development of osteosarcoma might proceed and related functions. The findings revealed that pyroptosis-related lncRNA signature was significantly enriched in natural killer cell mediated cytotoxicity, T-cell receptor signaling pathway, NOD-like receptor signaling pathway, complement and coagulation cascades, amino sugar and nucleotide sugar metabolism, apoptosis, primary immunodeficiency, and apoptosis in the low-risk group (Figure 7). A number of important insights were gained from our study, which will be used in future research to find novel personalized therapies and to accomplish full-process management of osteosarcoma patients from various risk categories.

### 3.8. Gene Expression and Immunity

**Figure 8:**
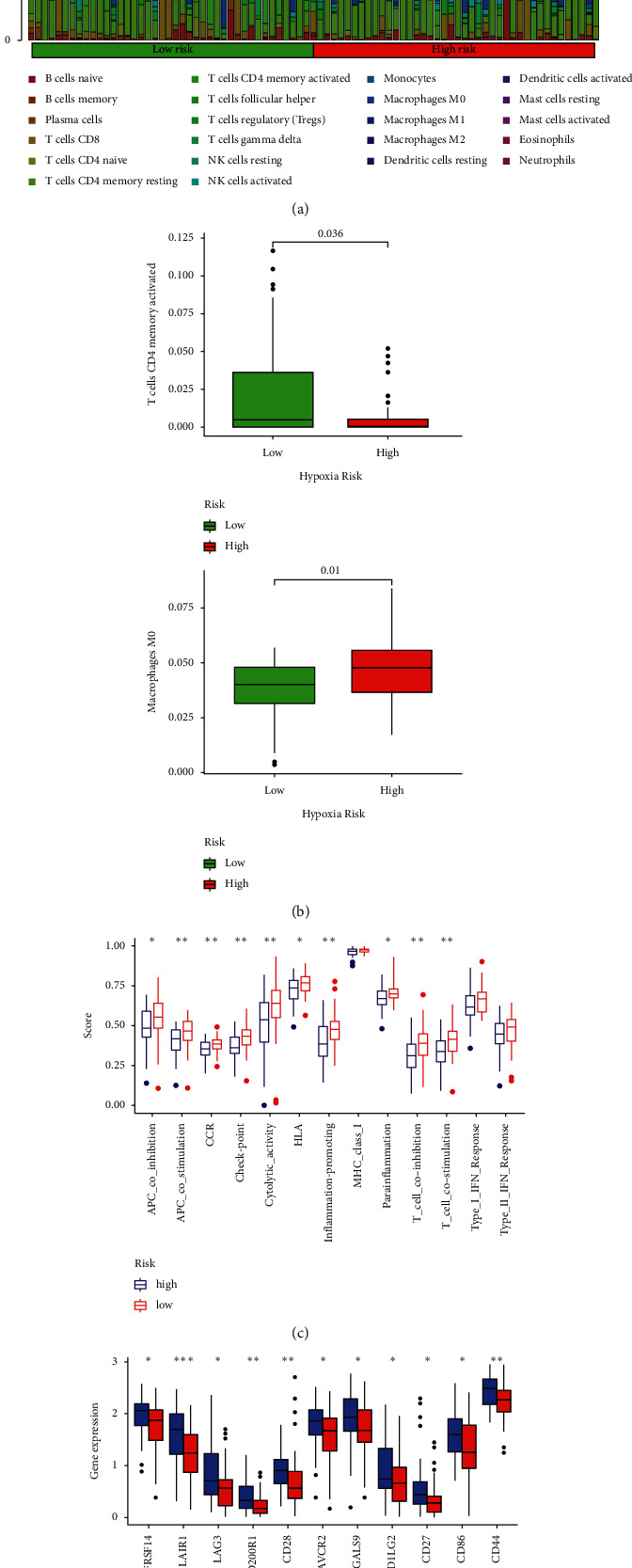
A tumor-infiltrating immune cell correlation with the risk model. (a, b) Heatmap and barplot of tumor-infiltrating immune cell types in low- and high-risk groups. (c) Immune-related activities in high- and low-risk groups based on ssGSEA. (d) Differences in immune checkpoint expression between high- and low-risk groups.

The CIBERSORT technique, in conjunction with the LM22 signature matrix, has been used to evaluate differences between low-risk and high-risk osteosarcoma patients in the infiltration of 22 distinct immune cell types.  Figure 8(a) summarizes the findings from TARGET's 88 osteosarcoma patients. The percentage of macrophage M0 cells was greater in high-risk patients, whereas the fraction of CD4 memory activated T cells was lower (Figure 8(b)). APC coinhibition and costimulation, CCR, checkpoint, cytolytic activity, HLA, and T-cell coinhibition and costimulation were significantly different between the low-risk and high-risk groups, according to a correlation analysis based on ssGSEA of TARGET data (Figure 8(c)). Given the significance of immunological checkpoints in both populations, between the two groups of patients, we discovered a significant variation in the expression of LAIR1, LAG3, CD44, and CD22 (Figure 8(d)).

## 4. Discussion

Patients with osteosarcoma continue to have a poor prognosis despite the fact that aggressive multidisciplinary treatment (surgery, radiation, chemotherapy, and immunotherapy) has significantly improved survival rates [20]. A patient's prognosis and treatment result may vary significantly even if they have the same clinical risk factors. It is therefore essential to find effective therapeutic targets for the diagnosis and treatment of osteosarcoma. Pyroptosis has been discovered to have a dual function in developing tumors and in treating processes throughout recent years as a new type of programmed cell death [21]. Promotion of tumor cell pyroptosis, on the other hand, may represent a novel therapeutic target. In this research, we utilized the TARGET database and STRING to examine variations in the expression of 33 pyroptosis-related genes in osteosarcoma, as well as protein interactions. To the best of our knowledge, this is the first comprehensive investigation of the involvement of pyroptosis in the development of osteosarcoma.

LncRNAs are abundantly produced in human cells, where they play an important role in a variety of biological processes, including genome expression and cell differentiation [22, 23]. Increasing evidence suggests that aberrant lncRNA expression may be associated with the incidence and progression of a number of malignancies. Currently, there is little research on pyroptosis-related lncRNAs. Recent studies have shown that XIST may suppress the development of non-small cell lung cancer by activating the miR-335/SOD2/ROS signaling pathway [24]. This signaling pathway leads to cell death by pyroptosis through the elongation of XIST, a long noncoding RNA (lncRNA) [25]. However, no studies have been conducted to investigate the function of pyroptosis-related lncRNA in osteosarcoma.

In this study, we examined the connection between 329 lncRNAs associated with pyroptosis and the forecasts of osteosarcoma. Furthermore, three pyroptosis-related lncRNAs of prognostic significance were discovered. We established a risk score to divide osteosarcoma patients into high- and low-risk groups and found that general survival rates were significantly different. A further finding is that a risk score based on the expression of three pyroptosis-related lncRNAs may accurately predict patient prognosis regardless of the presence or absence of traditional clinical risk markers or molecular features. Current risk scoring techniques for osteosarcoma rely mostly on whole genome sequencing. Previous research on the potential of lncRNAs as new tumor biomarkers has concentrated on individual molecules. As a new kind of malignant tumor biomarker, a lncRNA is not enough to use. As the lncRNAs are demonstrated to be secreted in a range of body fluids such as serum, saliva, and urine, the conclusion that lncRNAs are available in human serum/plasma seems plausible. Many research studies revealed the differential expression as a new kind of biomarker for patient evaluation of long noncoding RNAs in serum/plasma [26]. However, this is the first study to provide a risk sampling model for osteosarcoma based on three long RNAs with prognostic relevance linked to pyroptosis. Bioinformatics study has previously shown RPARP-AS1 and AC124312.3 in breast cancer [27, 28].

LncRNAs are also known to be involved in the immune microenvironment of tumors. The importance of immune-related lncRNAs has been shown in a variety of cancers [29, 30]. We examined the connection between tumor-infiltrating immune cells and the risk score in this study. The risk score was shown to be adversely associated with immune function and numerous immunological checkpoints. This research, therefore, is the first to examine the link between pyroptosis-related lncRNAs and tumor immunity in osteosarcoma.

However, we acknowledge that this research has significant limitations. The dataset utilized for the first study, for example, was inadequate. Because we obtained data only through TARGET, we were unable to obtain data on expression levels of other lncRNAs that support osteosarcoma, clinicopathological characteristics of patients, survival rates, and followup. To increase the reliability of the prediction findings, it is necessary to verify the newly built risk score model using tissue level and in vitro and in vivo studies. Overall, additional validation is necessary for the prediction model established in this research.

## 5. Conclusion

The prognostic model we have developed has been shown to have independent predictive value and great reliability and may provide some insight into future studies of lncRNA pyroptosis-related mechanisms, as well as new resources to better understand the immune cell specific gene mechanism involved in cancer control.

## Data Availability

The data used to support this study are available publicly in Therapeutically Applicable Research to Generate Effective Treatments (TARGET) and Genotype Tissue Expression (GTEx).

## Abbreviations

DEGs: Differentially expressed genes
TARGET: Therapeutically Applicable Research to Generate Effective Treatments
GTEx: Genotype Tissue Expression
GO: Gene Ontology
BP: Biological processes
MF: Molecular function
CC: Cellular components
KEGG: Kyoto Encyclopedia of Genes and Genomes
GSEA: Gene Set Enrichment Analyses
FDR: False discovery rate
ssGSEA: Single-Sample Gene Set Enrichment Analysis (ssGSEA).

## Supplementary Materials

Supplementary Materials
*Supplementary File Table S1*. 33 pyroptosis-related genes from prior reviews. *Supplementary File Table S2*. Patients' clinical features from the TARGET dataset. *Supplementary File Table S3*. Differential expression pyroptosis-related genes. *Supplementary File Table S4*. 329 pyroptosis-related lncRNAs by performing Pearson correlation analysis. *Supplementary File Figure S1*. The relationship between the novel lncRNA and mRNA.
