# Supplementary material for: Prognostic Value of a Pyroptosis-Related Long Noncoding RNA Signature Associated with Osteosarcoma Microenvironment
Source: J Oncol. 2021 Nov 11;2021:2182761. doi: 10.1155/2021/2182761 (PMC8601829; doi:10.1155/2021/2182761)
Supplement: Supplementary Materials — Supplementary File Table S1. 33 pyroptosis-related genes from prior reviews. Supplementary File Table S2. Patients' clinical features from the TARGET dataset. Supplementary File Table S3. Differential expression pyroptosis-related genes. Supplementary File Table S4. 329 pyroptosis-related lncRNAs by performing Pearson correlation analysis. Supplementary File Figure S1. The relationship between the novel lncRNA and mRNA. [file 2182761.f1.zip › 2182761.f1/Table S1.docx]

**Table S1:** 33 pyroptosis-related genes from prior reviews

AIM2

CASP1

CASP3

CASP4

CASP5

CASP6

CASP8

CASP9

ELANE

GPX4

GSDMA

GSDMB

GSDMC

GSDMD

GSDME

IL18

IL1B

IL6

NLRC4

NLRP1

NLRP2

NLRP3

NLRP6

NLRP7

NOD1

NOD2

PJVK

PLCG1

PRKACA

PYCARD

SCAF11

TIRAP

TNF
