# Supplementary material for: Prognostic Value of a Pyroptosis-Related Long Noncoding RNA Signature Associated with Osteosarcoma Microenvironment
Source: J Oncol. 2021 Nov 11;2021:2182761. doi: 10.1155/2021/2182761 (PMC8601829; doi:10.1155/2021/2182761)
Supplement: Supplementary Materials — Supplementary File Table S1. 33 pyroptosis-related genes from prior reviews. Supplementary File Table S2. Patients' clinical features from the TARGET dataset. Supplementary File Table S3. Differential expression pyroptosis-related genes. Supplementary File Table S4. 329 pyroptosis-related lncRNAs by performing Pearson correlation analysis. Supplementary File Figure S1. The relationship between the novel lncRNA and mRNA. [file 2182761.f1.zip › 2182761.f1/Table S2 (1).docx]

Table S2. Patients' clinical features from the TARGET dataset.

| **Variable** | **Number of samples** |
| --- | --- |
| **Gender** |  |
| Male | 48 |
| Female | 40 |
| **Age at diagnosis** |  |
| ≤14 | 41 |
| >14 | 47 |
| **Vital status** |  |
| Alive | 59 |
| Dead | 29 |
| **Metastatic** |  |
| No | 22 |
| Yes | 66 |
