# Supplementary material for: Prognostic Value of a Pyroptosis-Related Long Noncoding RNA Signature Associated with Osteosarcoma Microenvironment
Source: J Oncol. 2021 Nov 11;2021:2182761. doi: 10.1155/2021/2182761 (PMC8601829; doi:10.1155/2021/2182761)
Supplement: Supplementary Materials — Supplementary File Table S1. 33 pyroptosis-related genes from prior reviews. Supplementary File Table S2. Patients' clinical features from the TARGET dataset. Supplementary File Table S3. Differential expression pyroptosis-related genes. Supplementary File Table S4. 329 pyroptosis-related lncRNAs by performing Pearson correlation analysis. Supplementary File Figure S1. The relationship between the novel lncRNA and mRNA. [file 2182761.f1.zip › 2182761.f1/Table S3.docx]

Table S3. Differential expression pyroptosis-related genes.

| gene | nonMean | tumorMean | logFC | pValue | fdr |
| --- | --- | --- | --- | --- | --- |
| PRKACA | 6.615536 | 4.842251 | -1.77329 | 3.27E-48 | 2.48E-47 |
| NLRP1 | 2.353324 | 1.156344 | -1.19698 | 2.83E-35 | 6.27E-35 |
| GSDMC | 1.248803 | 0.100486 | -1.14832 | 1.81E-38 | 4.5E-38 |
| PJVK | 1.239616 | 0.245185 | -0.99443 | 3.51E-47 | 1.89E-46 |
| SCAF11 | 4.122565 | 3.219017 | -0.90355 | 4.58E-40 | 1.22E-39 |
| IL6 | 1.225956 | 0.357913 | -0.86804 | 5.11E-28 | 9.25E-28 |
| GSDME | 2.221438 | 1.378107 | -0.84333 | 4.74E-20 | 7.29E-20 |
| CASP9 | 2.450242 | 1.612077 | -0.83817 | 9.12E-32 | 1.81E-31 |
| GSDMB | 1.489244 | 0.776548 | -0.7127 | 2.47E-27 | 4.39E-27 |
| IL18 | 1.821882 | 1.140732 | -0.68115 | 3.49E-13 | 4.72E-13 |
| NOD1 | 1.570657 | 1.037946 | -0.53271 | 1.38E-24 | 2.32E-24 |
| ELANE | 0.606081 | 0.230437 | -0.57564 | 1.43E-25 | 2.45E-25 |
| PLCG1 | 4.224147 | 3.920817 | -0.50333 | 5.25E-07 | 6.31E-07 |
| GPX4 | 7.610248 | 7.400024 | -0.51022 | 0.00505 | 0.005507 |
| TIRAP | 1.394685 | 1.667739 | 0.573054 | 2.37E-07 | 2.88E-07 |
| CASP8 | 1.080499 | 1.413791 | 0.533291 | 6.21E-09 | 7.76E-09 |
| GSDMD | 2.937276 | 3.540144 | 0.602868 | 1.29E-11 | 1.69E-11 |
| PYCARD | 1.211433 | 2.322397 | 1.110964 | 2.09E-20 | 3.24E-20 |
| CASP6 | 1.586316 | 2.809329 | 1.223013 | 1.9E-41 | 5.46E-41 |
| CASP3 | 2.538809 | 4.060804 | 1.521995 | 5.44E-43 | 1.73E-42 |
