# Supplementary material for: Prognostic Value of a Pyroptosis-Related Long Noncoding RNA Signature Associated with Osteosarcoma Microenvironment
Source: J Oncol. 2021 Nov 11;2021:2182761. doi: 10.1155/2021/2182761 (PMC8601829; doi:10.1155/2021/2182761)
Supplement: Supplementary Materials — Supplementary File Table S1. 33 pyroptosis-related genes from prior reviews. Supplementary File Table S2. Patients' clinical features from the TARGET dataset. Supplementary File Table S3. Differential expression pyroptosis-related genes. Supplementary File Table S4. 329 pyroptosis-related lncRNAs by performing Pearson correlation analysis. Supplementary File Figure S1. The relationship between the novel lncRNA and mRNA. [file 2182761.f1.zip › 2182761.f1/Table S4.docx]

Table S4. 329 pyroptosis-related lncRNAs by performing Pearson correlation analysis

| ferrGene | lncRNA | cor | pvalue | Regulation |
| --- | --- | --- | --- | --- |
| IL6 | AC005696.3 | 0.513154 | 3.19E-07 | postive |
| CASP8 | AC009237.15 | 0.43935 | 1.85E-05 | postive |
| PRKACA | FOXD2-AS1 | 0.468051 | 4.26E-06 | postive |
| IL6 | AL662844.3 | 0.503859 | 5.61E-07 | postive |
| GSDME | AC006042.1 | 0.408052 | 7.94E-05 | postive |
| SCAF11 | AL023803.1 | -0.43364 | 2.44E-05 | negative |
| ELANE | AC092611.1 | 0.426438 | 3.43E-05 | postive |
| CASP9 | MYLK-AS1 | 0.522425 | 1.78E-07 | postive |
| IL6 | AL513477.2 | 0.426291 | 3.46E-05 | postive |
| AIM2 | AC087286.2 | 0.425281 | 3.62E-05 | postive |
| NOD1 | AC087286.2 | 0.435674 | 2.21E-05 | postive |
| IL6 | STXBP5-AS1 | 0.536399 | 7.18E-08 | postive |
| GSDMD | AC124312.2 | -0.4009 | 0.000109 | negative |
| AIM2 | LINC01615 | 0.435977 | 2.18E-05 | postive |
| CASP1 | AC022034.1 | 0.412669 | 6.46E-05 | postive |
| CASP4 | AC131934.1 | -0.46054 | 6.33E-06 | negative |
| NOD2 | AC010809.2 | 0.503707 | 5.66E-07 | postive |
| PJVK | AC010809.2 | 0.515196 | 2.81E-07 | postive |
| IL6 | BX640514.2 | 0.434058 | 2.39E-05 | postive |
| AIM2 | LINC01711 | 0.549384 | 2.97E-08 | postive |
| IL6 | AC079148.1 | 0.490425 | 1.23E-06 | postive |
| IL6 | LINC02613 | 0.400357 | 0.000111 | postive |
| AIM2 | AC069360.1 | 0.567771 | 7.98E-09 | postive |
| GSDME | AC069360.1 | 0.488148 | 1.40E-06 | postive |
| GSDMB | DNAJB5-DT | 0.414315 | 6.00E-05 | postive |
| GSDMC | AC073389.3 | 0.433211 | 2.49E-05 | postive |
| NOD2 | AC092368.3 | 0.423697 | 3.90E-05 | postive |
| IL6 | LUCAT1 | 0.563264 | 1.11E-08 | postive |
| CASP4 | LINC01836 | 0.465011 | 5.01E-06 | postive |
| CASP1 | AC009275.1 | 0.401139 | 0.000107 | postive |
| SCAF11 | HDAC4-AS1 | -0.43461 | 2.33E-05 | negative |
| AIM2 | AL391807.1 | -0.42131 | 4.36E-05 | negative |
| IL6 | AL355312.2 | 0.61193 | 2.39E-10 | postive |
| CASP3 | AC138230.1 | -0.4053 | 8.96E-05 | negative |
| AIM2 | LINC00662 | -0.46815 | 4.24E-06 | negative |
| PRKACA | LINC00662 | -0.45342 | 9.15E-06 | negative |
| CASP6 | LINC00654 | 0.414796 | 5.87E-05 | postive |
| NOD2 | AC023906.5 | 0.439941 | 1.80E-05 | postive |
| PJVK | AC023906.5 | 0.446344 | 1.31E-05 | postive |
| IL6 | SNHG22 | 0.4472 | 1.25E-05 | postive |
| IL6 | AC092757.3 | 0.451411 | 1.01E-05 | postive |
| NLRC4 | AC080112.1 | 0.426117 | 3.48E-05 | postive |
| IL6 | LAMC1-AS1 | 0.430187 | 2.87E-05 | postive |
| GSDMB | AC091965.1 | 0.414334 | 5.99E-05 | postive |
| GSDMC | AL139384.1 | 0.632904 | 3.70E-11 | postive |
| CASP1 | AC138207.5 | 0.670683 | 8.77E-13 | postive |
| IL18 | AC138207.5 | 0.681297 | 2.77E-13 | postive |
| NLRC4 | AC138207.5 | 0.635138 | 3.01E-11 | postive |
| NLRP3 | AC138207.5 | 0.756592 | 1.53E-17 | postive |
| PYCARD | AC138207.5 | 0.704937 | 1.78E-14 | postive |
| AIM2 | LINC02298 | -0.40963 | 7.40E-05 | negative |
| SCAF11 | AL133260.1 | -0.41031 | 7.18E-05 | negative |
| IL6 | AP001107.1 | 0.407093 | 8.28E-05 | postive |
| SCAF11 | LINC00265 | 0.401965 | 0.000104 | postive |
| GSDMD | AL441992.1 | 0.406764 | 8.40E-05 | postive |
| GSDMC | AC019186.1 | 0.404922 | 9.11E-05 | postive |
| NLRP1 | LINC00641 | 0.413057 | 6.35E-05 | postive |
| IL6 | AP001542.3 | 0.487629 | 1.45E-06 | postive |
| IL6 | AP000866.2 | 0.436733 | 2.10E-05 | postive |
| IL6 | AC005014.2 | 0.433518 | 2.45E-05 | postive |
| AIM2 | AP003071.4 | 0.431337 | 2.72E-05 | postive |
| AIM2 | MSC-AS1 | 0.465685 | 4.83E-06 | postive |
| GSDME | MSC-AS1 | 0.553289 | 2.26E-08 | postive |
| NLRP1 | MSC-AS1 | 0.469964 | 3.84E-06 | postive |
| NOD1 | MSC-AS1 | 0.452673 | 9.51E-06 | postive |
| GSDMD | AC005520.2 | 0.407789 | 8.03E-05 | postive |
| GSDMC | AC021739.2 | 0.670158 | 9.27E-13 | postive |
| CASP8 | AP005329.2 | 0.426418 | 3.43E-05 | postive |
| ELANE | SOCS2-AS1 | 0.455545 | 8.21E-06 | postive |
| NLRP3 | LINC01819 | 0.409969 | 7.29E-05 | postive |
| AIM2 | KDM7A-DT | 0.432354 | 2.59E-05 | postive |
| GSDME | KDM7A-DT | 0.45081 | 1.05E-05 | postive |
| GSDMD | AL117332.1 | 0.425375 | 3.61E-05 | postive |
| CASP1 | PCED1B-AS1 | 0.64714 | 9.60E-12 | postive |
| GSDMD | PCED1B-AS1 | 0.406304 | 8.57E-05 | postive |
| IL18 | PCED1B-AS1 | 0.628245 | 5.66E-11 | postive |
| NLRC4 | PCED1B-AS1 | 0.657324 | 3.50E-12 | postive |
| NLRP3 | PCED1B-AS1 | 0.638486 | 2.20E-11 | postive |
| PYCARD | PCED1B-AS1 | 0.819888 | 1.54E-22 | postive |
| TIRAP | AP001318.2 | 0.628351 | 5.61E-11 | postive |
| SCAF11 | AC093726.1 | 0.400513 | 0.00011 | postive |
| GPX4 | AC005288.1 | -0.40397 | 9.50E-05 | negative |
| SCAF11 | AC005288.1 | 0.460608 | 6.31E-06 | postive |
| IL6 | AJ011932.1 | 0.456521 | 7.80E-06 | postive |
| PYCARD | AC069281.2 | 0.443643 | 1.50E-05 | postive |
| AIM2 | LINC00565 | 0.420533 | 4.52E-05 | postive |
| IL6 | LINC00565 | 0.555129 | 1.99E-08 | postive |
| CASP1 | AC108449.2 | -0.41036 | 7.16E-05 | negative |
| PYCARD | AC108449.2 | -0.50396 | 5.57E-07 | negative |
| SCAF11 | AC108449.2 | 0.42326 | 3.98E-05 | postive |
| GSDME | AC093673.1 | 0.420145 | 4.60E-05 | postive |
| GSDME | GAPLINC | 0.460222 | 6.44E-06 | postive |
| PYCARD | GAPLINC | 0.438721 | 1.91E-05 | postive |
| CASP9 | AC011477.2 | -0.41296 | 6.38E-05 | negative |
| GSDMC | AC063948.1 | 0.798287 | 1.23E-20 | postive |
| GSDMC | AC004076.2 | 0.560255 | 1.38E-08 | postive |
| NOD2 | AC053503.4 | 0.533172 | 8.89E-08 | postive |
| GSDME | AC092747.4 | 0.420028 | 4.62E-05 | postive |
| SCAF11 | RARA-AS1 | -0.40404 | 9.47E-05 | negative |
| NLRP3 | NUP50-DT | 0.414511 | 5.94E-05 | postive |
| PYCARD | NUP50-DT | 0.454961 | 8.46E-06 | postive |
| NOD2 | AL137782.1 | 0.415586 | 5.66E-05 | postive |
| SCAF11 | AC024075.1 | 0.430099 | 2.89E-05 | postive |
| PJVK | AC105285.1 | 0.412337 | 6.56E-05 | postive |
| GSDMB | AC091965.4 | 0.44481 | 1.41E-05 | postive |
| GSDMC | HOTAIR | 0.40851 | 7.78E-05 | postive |
| IL6 | AL731563.3 | 0.451511 | 1.01E-05 | postive |
| GSDMC | CRNDE | 0.42126 | 4.37E-05 | postive |
| NOD1 | SH3RF3-AS1 | 0.463303 | 5.48E-06 | postive |
| AIM2 | LINC01549 | -0.42934 | 2.99E-05 | negative |
| GSDME | LINC01549 | -0.44271 | 1.57E-05 | negative |
| AIM2 | AC010609.1 | -0.42115 | 4.39E-05 | negative |
| NOD1 | AL355001.2 | 0.409828 | 7.33E-05 | postive |
| CASP1 | WDR86-AS1 | 0.421824 | 4.25E-05 | postive |
| PYCARD | WDR86-AS1 | 0.407121 | 8.27E-05 | postive |
| GSDMB | SCGB1B2P | 0.40938 | 7.48E-05 | postive |
| GPX4 | ZNF236-DT | 0.412916 | 6.39E-05 | postive |
| CASP1 | AL590226.1 | 0.42255 | 4.11E-05 | postive |
| IL18 | AL590226.1 | 0.440732 | 1.73E-05 | postive |
| PYCARD | AL590226.1 | 0.420489 | 4.52E-05 | postive |
| CASP1 | AL928654.1 | -0.40179 | 0.000104 | negative |
| PYCARD | AL928654.1 | -0.42163 | 4.29E-05 | negative |
| NLRP1 | STARD4-AS1 | 0.413241 | 6.29E-05 | postive |
| SCAF11 | STARD4-AS1 | 0.406463 | 8.51E-05 | postive |
| IL6 | AC104463.2 | 0.534652 | 8.07E-08 | postive |
| CASP4 | AC019205.1 | -0.44578 | 1.35E-05 | negative |
| SCAF11 | AC009118.2 | -0.43732 | 2.04E-05 | negative |
| IL6 | GAS6-AS1 | 0.517968 | 2.36E-07 | postive |
| CASP3 | AC008731.1 | -0.40531 | 8.95E-05 | negative |
| IL6 | AC008731.1 | 0.632725 | 3.76E-11 | postive |
| AIM2 | AL355916.1 | 0.457966 | 7.24E-06 | postive |
| IL6 | AL355916.1 | 0.421233 | 4.37E-05 | postive |
| ELANE | AC063943.1 | 0.414943 | 5.83E-05 | postive |
| GSDME | AC097359.2 | 0.445164 | 1.39E-05 | postive |
| AIM2 | AC022509.3 | 0.472137 | 3.42E-06 | postive |
| IL6 | CBR3-AS1 | 0.449846 | 1.10E-05 | postive |
| GSDMC | AC103691.1 | 0.425183 | 3.64E-05 | postive |
| GSDMC | ZKSCAN2-DT | 0.728299 | 8.92E-16 | postive |
| GSDMD | LINC01770 | 0.431533 | 2.70E-05 | postive |
| ELANE | TUBA3FP | 0.472167 | 3.41E-06 | postive |
| CASP3 | AC074029.3 | -0.42671 | 3.39E-05 | negative |
| IL6 | AC074029.3 | 0.618377 | 1.37E-10 | postive |
| IL18 | ZBED5-AS1 | 0.458505 | 7.04E-06 | postive |
| PYCARD | ZBED5-AS1 | 0.412204 | 6.59E-05 | postive |
| NLRP3 | AC026369.2 | 0.472185 | 3.41E-06 | postive |
| PYCARD | AC026369.2 | 0.408097 | 7.92E-05 | postive |
| PYCARD | LINC00324 | 0.405983 | 8.69E-05 | postive |
| IL6 | AL035530.2 | 0.434891 | 2.30E-05 | postive |
| ELANE | HGC6.3 | 0.54867 | 3.12E-08 | postive |
| GSDMC | AC009269.5 | 0.463973 | 5.29E-06 | postive |
| IL6 | HID1-AS1 | 0.53274 | 9.15E-08 | postive |
| GSDMB | AP001160.1 | 0.420744 | 4.47E-05 | postive |
| AIM2 | AP002884.1 | -0.44142 | 1.67E-05 | negative |
| GSDMB | AC009126.1 | 0.445537 | 1.36E-05 | postive |
| CASP1 | AC116407.2 | 0.43687 | 2.09E-05 | postive |
| CASP1 | AC124319.1 | 0.436605 | 2.11E-05 | postive |
| CASP8 | AC124319.1 | 0.403774 | 9.58E-05 | postive |
| GSDME | LINC00852 | 0.444557 | 1.43E-05 | postive |
| SCAF11 | AC048382.2 | 0.403939 | 9.51E-05 | postive |
| CASP8 | AP003352.1 | -0.44099 | 1.71E-05 | negative |
| GSDMB | AL162586.1 | 0.541327 | 5.16E-08 | postive |
| AIM2 | LINC01060 | -0.42691 | 3.36E-05 | negative |
| PYCARD | RASSF1-AS1 | 0.473548 | 3.17E-06 | postive |
| NLRP1 | AC010889.1 | 0.420883 | 4.44E-05 | postive |
| ELANE | AC008393.1 | 0.442135 | 1.61E-05 | postive |
| GSDMC | AC090246.1 | 0.572239 | 5.73E-09 | postive |
| CASP8 | AC093388.1 | 0.463051 | 5.55E-06 | postive |
| IL6 | AL591178.1 | 0.597111 | 8.23E-10 | postive |
| CASP4 | AC010834.3 | -0.52704 | 1.33E-07 | negative |
| CASP9 | AC006449.6 | 0.404193 | 9.40E-05 | postive |
| PYCARD | AC008556.1 | 0.417681 | 5.15E-05 | postive |
| GSDMC | AC016396.1 | 0.43278 | 2.54E-05 | postive |
| CASP1 | AC009950.1 | 0.417982 | 5.08E-05 | postive |
| GSDMC | PSMD6-AS2 | 0.797489 | 1.44E-20 | postive |
| GSDMB | AC132872.1 | 0.444064 | 1.47E-05 | postive |
| NLRP1 | ACTA2-AS1 | 0.434237 | 2.37E-05 | postive |
| GSDMC | AP001767.2 | 0.544209 | 4.25E-08 | postive |
| ELANE | ZNF561-AS1 | 0.458809 | 6.93E-06 | postive |
| IL6 | LINC01359 | 0.489456 | 1.30E-06 | postive |
| NLRP1 | TRAM2-AS1 | 0.407286 | 8.21E-05 | postive |
| SCAF11 | AL132989.1 | 0.404249 | 9.38E-05 | postive |
| CASP8 | LPP-AS2 | 0.448336 | 1.18E-05 | postive |
| GSDMC | AL136141.1 | 0.671184 | 8.31E-13 | postive |
| AIM2 | MIR155HG | 0.547673 | 3.35E-08 | postive |
| GSDMC | RNFT1-DT | 0.603744 | 4.77E-10 | postive |
| ELANE | AC099778.1 | 0.408405 | 7.81E-05 | postive |
| GSDMC | AC135050.3 | 0.565798 | 9.23E-09 | postive |
| GSDMC | RFX3-AS1 | 0.566785 | 8.59E-09 | postive |
| IL6 | ELF3-AS1 | 0.472215 | 3.40E-06 | postive |
| AIM2 | AP000695.2 | 0.481235 | 2.07E-06 | postive |
| GSDME | AP000695.2 | 0.53381 | 8.53E-08 | postive |
| ELANE | BDNF-AS | 0.435222 | 2.26E-05 | postive |
| SCAF11 | AC016747.3 | 0.411157 | 6.91E-05 | postive |
| PYCARD | AC137630.3 | 0.442911 | 1.55E-05 | postive |
| PYCARD | AC109322.1 | 0.400884 | 0.000109 | postive |
| ELANE | GRIK1-AS1 | 0.410138 | 7.23E-05 | postive |
| IL6 | MIR222HG | 0.513809 | 3.06E-07 | postive |
| GSDMC | AC060766.5 | 0.587386 | 1.79E-09 | postive |
| TIRAP | AC009948.1 | -0.40495 | 9.10E-05 | negative |
| IL6 | AC010973.1 | 0.580832 | 2.99E-09 | postive |
| SCAF11 | Z68871.1 | 0.409937 | 7.30E-05 | postive |
| SCAF11 | AL121832.2 | -0.45766 | 7.36E-06 | negative |
| AIM2 | AL356481.3 | 0.435408 | 2.24E-05 | postive |
| AIM2 | BBOX1-AS1 | 0.619643 | 1.22E-10 | postive |
| GSDMC | AC007546.1 | 0.461534 | 6.01E-06 | postive |
| PRKACA | AC093752.1 | -0.40623 | 8.60E-05 | negative |
| GSDME | AC005229.4 | 0.443461 | 1.51E-05 | postive |
| CASP6 | AL049838.1 | 0.432936 | 2.52E-05 | postive |
| NLRP1 | GHRLOS | 0.411516 | 6.80E-05 | postive |
| SCAF11 | AC011477.3 | 0.460669 | 6.29E-06 | postive |
| CASP6 | AC234031.1 | 0.400183 | 0.000112 | postive |
| CASP1 | AC138207.4 | 0.481997 | 1.99E-06 | postive |
| IL18 | AC138207.4 | 0.490919 | 1.20E-06 | postive |
| NLRC4 | AC138207.4 | 0.465826 | 4.80E-06 | postive |
| NLRP3 | AC138207.4 | 0.679707 | 3.30E-13 | postive |
| PYCARD | AC138207.4 | 0.512664 | 3.28E-07 | postive |
| CASP3 | RBPMS-AS1 | -0.41095 | 6.98E-05 | negative |
| IL6 | RBPMS-AS1 | 0.600079 | 6.45E-10 | postive |
| PYCARD | AC006213.1 | -0.40291 | 9.94E-05 | negative |
| CASP1 | AL357033.4 | 0.538792 | 6.12E-08 | postive |
| NLRC4 | AL357033.4 | 0.47099 | 3.64E-06 | postive |
| NLRP3 | AL357033.4 | 0.430795 | 2.79E-05 | postive |
| PYCARD | AL357033.4 | 0.444139 | 1.46E-05 | postive |
| CASP1 | TP53TG1 | 0.429238 | 3.01E-05 | postive |
| CASP4 | AL139393.2 | -0.40367 | 9.62E-05 | negative |
| SCAF11 | SNHG9 | -0.52608 | 1.41E-07 | negative |
| GSDMB | AP000757.1 | 0.4089 | 7.64E-05 | postive |
| AIM2 | LINC00327 | 0.421272 | 4.36E-05 | postive |
| GPX4 | LINC00327 | -0.4217 | 4.28E-05 | negative |
| ELANE | LINC00989 | 0.423556 | 3.93E-05 | postive |
| GSDMC | AC107871.2 | 0.823597 | 6.84E-23 | postive |
| IL6 | AL158151.4 | 0.439228 | 1.86E-05 | postive |
| CASP1 | AC147067.2 | 0.471757 | 3.49E-06 | postive |
| IL18 | AC147067.2 | 0.438764 | 1.90E-05 | postive |
| NLRC4 | AC147067.2 | 0.453357 | 9.18E-06 | postive |
| PYCARD | AC147067.2 | 0.441795 | 1.64E-05 | postive |
| IL6 | AC104938.1 | 0.612729 | 2.23E-10 | postive |
| GSDMC | LOXL1-AS1 | 0.518933 | 2.22E-07 | postive |
| CASP9 | AP001453.2 | 0.425316 | 3.62E-05 | postive |
| GSDME | AP001453.2 | 0.426261 | 3.46E-05 | postive |
| GSDMC | AL591895.1 | 0.501919 | 6.29E-07 | postive |
| GSDMB | AC009812.4 | 0.400765 | 0.000109 | postive |
| PRKACA | AC006504.1 | -0.44794 | 1.21E-05 | negative |
| NOD2 | LINC02308 | 0.498053 | 7.91E-07 | postive |
| PJVK | LINC02308 | 0.508454 | 4.25E-07 | postive |
| AIM2 | AC073611.1 | -0.53534 | 7.71E-08 | negative |
| GSDME | AC073611.1 | -0.46079 | 6.25E-06 | negative |
| NLRP1 | AC073611.1 | -0.41038 | 7.15E-05 | negative |
| GSDMC | HAGLROS | 0.471415 | 3.55E-06 | postive |
| GSDMC | AC005920.4 | 0.435414 | 2.24E-05 | postive |
| CASP6 | AC022007.1 | 0.400358 | 0.000111 | postive |
| AIM2 | AL645608.6 | -0.42932 | 3.00E-05 | negative |
| GSDME | AL645608.6 | -0.4998 | 7.14E-07 | negative |
| PYCARD | AL035446.1 | 0.498645 | 7.64E-07 | postive |
| AIM2 | DLGAP1-AS2 | 0.42629 | 3.46E-05 | postive |
| NOD2 | BVES-AS1 | 0.427411 | 3.28E-05 | postive |
| PJVK | BVES-AS1 | 0.404614 | 9.23E-05 | postive |
| AIM2 | APCDD1L-DT | 0.421353 | 4.35E-05 | postive |
| GSDME | APCDD1L-DT | 0.452979 | 9.36E-06 | postive |
| IL6 | AL049780.2 | 0.560944 | 1.31E-08 | postive |
| CASP1 | AC145098.1 | 0.422805 | 4.07E-05 | postive |
| IL18 | AC145098.1 | 0.464286 | 5.20E-06 | postive |
| NLRC4 | AC145098.1 | 0.514415 | 2.95E-07 | postive |
| PYCARD | AC145098.1 | 0.444787 | 1.41E-05 | postive |
| GSDME | RNF216P1 | 0.496205 | 8.82E-07 | postive |
| NLRP1 | CARMN | 0.457297 | 7.50E-06 | postive |
| AIM2 | LINC00511 | 0.491807 | 1.14E-06 | postive |
| PYCARD | AL121894.2 | 0.42026 | 4.57E-05 | postive |
| CASP1 | AC108134.3 | 0.452563 | 9.56E-06 | postive |
| IL18 | AC108134.3 | 0.510852 | 3.67E-07 | postive |
| PYCARD | AC108134.3 | 0.507225 | 4.58E-07 | postive |
| IL6 | AL596442.2 | 0.487749 | 1.44E-06 | postive |
| CASP1 | PSMB8-AS1 | 0.650471 | 6.93E-12 | postive |
| IL18 | PSMB8-AS1 | 0.462771 | 5.64E-06 | postive |
| NLRP3 | PSMB8-AS1 | 0.409816 | 7.34E-05 | postive |
| PYCARD | PSMB8-AS1 | 0.486583 | 1.53E-06 | postive |
| GSDMB | AC007032.1 | 0.413501 | 6.22E-05 | postive |
| PLCG1 | ZNF436-AS1 | 0.435185 | 2.26E-05 | postive |
| AIM2 | JMJD1C-AS1 | -0.41666 | 5.39E-05 | negative |
| IL6 | AL359962.1 | 0.536178 | 7.29E-08 | postive |
| CASP3 | AL139289.2 | -0.44708 | 1.26E-05 | negative |
| PYCARD | SERPINB9P1 | 0.434646 | 2.32E-05 | postive |
| IL6 | AC007666.1 | 0.615118 | 1.81E-10 | postive |
| IL6 | AL360091.1 | 0.555505 | 1.94E-08 | postive |
| CASP1 | PCAT19 | 0.493753 | 1.02E-06 | postive |
| CASP4 | PCAT19 | 0.457637 | 7.37E-06 | postive |
| CASP3 | AC080129.2 | -0.40217 | 0.000103 | negative |
| IL6 | AC080129.2 | 0.657324 | 3.50E-12 | postive |
| CASP3 | AC138811.1 | -0.40147 | 0.000106 | negative |
| IL6 | AC138811.1 | 0.585278 | 2.11E-09 | postive |
| GSDME | LINC01719 | -0.41724 | 5.25E-05 | negative |
| PYCARD | U62317.2 | 0.490122 | 1.25E-06 | postive |
| IL6 | AC107021.2 | 0.418481 | 4.96E-05 | postive |
| GSDMC | RPARP-AS1 | 0.587882 | 1.72E-09 | postive |
| GSDMC | AC012254.1 | 0.584606 | 2.23E-09 | postive |
| GSDMB | FOCAD-AS1 | 0.401181 | 0.000107 | postive |
| CASP1 | LINC00844 | 0.42505 | 3.66E-05 | postive |
| CASP1 | HCP5 | 0.438522 | 1.92E-05 | postive |
| IL18 | HCP5 | 0.431391 | 2.71E-05 | postive |
| NOD2 | HCP5 | 0.407213 | 8.24E-05 | postive |
| CASP4 | AC090152.1 | -0.40177 | 0.000105 | negative |
| AIM2 | AC092164.1 | -0.4492 | 1.13E-05 | negative |
| NOD2 | SCAT8 | 0.492045 | 1.12E-06 | postive |
| PJVK | SCAT8 | 0.580648 | 3.03E-09 | postive |
| CASP1 | AC008592.3 | 0.417367 | 5.22E-05 | postive |
| IL18 | AC008592.3 | 0.400427 | 0.000111 | postive |
| ELANE | AC118344.1 | 0.418199 | 5.03E-05 | postive |
| CASP3 | AP000345.2 | -0.42041 | 4.54E-05 | negative |
| IL6 | AP000345.2 | 0.587136 | 1.83E-09 | postive |
| CASP8 | LINC01614 | 0.408665 | 7.72E-05 | postive |
| SCAF11 | PCAT6 | -0.43283 | 2.53E-05 | negative |
| IL6 | AC005519.1 | 0.403418 | 9.73E-05 | postive |
| CASP8 | AL359076.1 | 0.442172 | 1.61E-05 | postive |
| CASP1 | AL031775.1 | 0.453573 | 9.08E-06 | postive |
| AIM2 | AP000695.1 | 0.469673 | 3.90E-06 | postive |
| GSDME | AP000695.1 | 0.421764 | 4.27E-05 | postive |
| NOD2 | AP000695.1 | 0.428668 | 3.09E-05 | postive |
| SCAF11 | PAXIP1-AS2 | 0.439393 | 1.84E-05 | postive |
| GPX4 | AC027307.2 | 0.449911 | 1.09E-05 | postive |
| PYCARD | PRR34-AS1 | 0.463411 | 5.45E-06 | postive |
| GSDMB | AC087741.1 | 0.502668 | 6.02E-07 | postive |
| GSDMB | FAM215B | 0.456498 | 7.81E-06 | postive |
| GSDME | RUSC1-AS1 | -0.40822 | 7.88E-05 | negative |
| IL6 | AC004241.2 | 0.407431 | 8.16E-05 | postive |
| PJVK | PAN3-AS1 | 0.403902 | 9.52E-05 | postive |
| GSDMC | AC004492.1 | 0.441962 | 1.63E-05 | postive |
| GSDMC | AC012442.1 | 0.435316 | 2.25E-05 | postive |
| GSDMC | AC105339.3 | 0.674274 | 5.97E-13 | postive |
| AIM2 | AL137186.2 | 0.48115 | 2.08E-06 | postive |
| SCAF11 | AC145343.1 | -0.41942 | 4.75E-05 | negative |
| CASP9 | ZNF687-AS1 | 0.427668 | 3.24E-05 | postive |
| GSDME | AC073115.1 | 0.547627 | 3.36E-08 | postive |
| NOD2 | AC073115.1 | 0.415402 | 5.71E-05 | postive |
| CASP3 | TENM3-AS1 | 0.400294 | 0.000111 | postive |
| PRKACA | AL596244.1 | 0.400413 | 0.000111 | postive |
| CASP3 | AL928921.1 | -0.44486 | 1.41E-05 | negative |
| IL6 | AL928921.1 | 0.615941 | 1.69E-10 | postive |
| PRKACA | AC024075.2 | 0.456089 | 7.98E-06 | postive |
| CASP1 | AC093278.2 | 0.613787 | 2.03E-10 | postive |
| IL18 | AC093278.2 | 0.580247 | 3.12E-09 | postive |
| NLRC4 | AC093278.2 | 0.451988 | 9.84E-06 | postive |
| NLRP3 | AC093278.2 | 0.458615 | 7.00E-06 | postive |
| PYCARD | AC093278.2 | 0.495591 | 9.14E-07 | postive |
| CASP1 | AC008972.2 | 0.474422 | 3.02E-06 | postive |
| IL18 | AC008972.2 | 0.522868 | 1.73E-07 | postive |
| PYCARD | AC008972.2 | 0.537323 | 6.75E-08 | postive |
| AIM2 | AC010457.1 | 0.427937 | 3.20E-05 | postive |
| AIM2 | LINC02593 | -0.56547 | 9.45E-09 | negative |
| GSDME | LINC02593 | -0.57358 | 5.18E-09 | negative |
| NOD2 | LINCMD1 | 0.482319 | 1.95E-06 | postive |
| PJVK | LINCMD1 | 0.426711 | 3.39E-05 | postive |
| CASP3 | AC131097.3 | -0.43955 | 1.83E-05 | negative |
| IL6 | AC131097.3 | 0.573161 | 5.35E-09 | postive |
| GSDMD | LINC01137 | 0.474794 | 2.96E-06 | postive |
| PYCARD | LINC01137 | 0.532821 | 9.10E-08 | postive |
| NOD2 | LINC02544 | 0.412866 | 6.40E-05 | postive |
| GSDMC | AC018926.1 | 0.617228 | 1.51E-10 | postive |
| CASP1 | AL161785.1 | 0.51674 | 2.55E-07 | postive |
| IL18 | AL161785.1 | 0.582758 | 2.57E-09 | postive |
| NLRC4 | AL161785.1 | 0.550281 | 2.79E-08 | postive |
| NLRP3 | AL161785.1 | 0.70718 | 1.35E-14 | postive |
| PYCARD | AL161785.1 | 0.663781 | 1.81E-12 | postive |
| CASP4 | SENCR | 0.406581 | 8.47E-05 | postive |
| GSDME | AC009318.2 | 0.439731 | 1.81E-05 | postive |
| CASP8 | Z83843.1 | 0.443663 | 1.50E-05 | postive |
| IL6 | AC012676.3 | 0.569604 | 6.97E-09 | postive |
| IL6 | AC069307.1 | 0.400565 | 0.00011 | postive |
| GSDMB | AC022167.2 | 0.406986 | 8.32E-05 | postive |
| GSDMC | CAPN10-DT | 0.722541 | 1.92E-15 | postive |
| AIM2 | C1RL-AS1 | 0.502458 | 6.09E-07 | postive |
| ELANE | AC022784.5 | 0.412945 | 6.38E-05 | postive |
| NLRP1 | AL137003.1 | 0.406818 | 8.38E-05 | postive |
| NOD1 | AL137003.1 | 0.461006 | 6.18E-06 | postive |
| AIM2 | AC104695.3 | 0.406589 | 8.46E-05 | postive |
| GSDME | AC104695.3 | 0.451123 | 1.03E-05 | postive |
| PYCARD | CYTOR | 0.421593 | 4.30E-05 | postive |
| GSDME | LINC01894 | 0.40577 | 8.78E-05 | postive |
| NOD2 | LINC01894 | 0.400184 | 0.000112 | postive |
| CASP3 | AL096701.3 | -0.42971 | 2.94E-05 | negative |
| IL6 | AL096701.3 | 0.588737 | 1.61E-09 | postive |
| IL6 | AC138150.1 | 0.604111 | 4.62E-10 | postive |
| IL6 | AC232271.1 | 0.401844 | 0.000104 | postive |
| PRKACA | AC022098.1 | 0.404691 | 9.20E-05 | postive |
| GSDMC | AC025165.4 | 0.401988 | 0.000104 | postive |
| GSDMC | AP002336.2 | 0.623972 | 8.32E-11 | postive |
| CASP1 | LINC01094 | 0.45501 | 8.44E-06 | postive |
| GSDME | LINC01094 | 0.435939 | 2.18E-05 | postive |
| IL18 | LINC01094 | 0.431544 | 2.69E-05 | postive |
| NLRC4 | LINC01094 | 0.536478 | 7.15E-08 | postive |
| NLRP3 | LINC01094 | 0.59039 | 1.41E-09 | postive |
| PYCARD | LINC01094 | 0.413197 | 6.31E-05 | postive |
| AIM2 | LINC01679 | 0.438396 | 1.94E-05 | postive |
| CASP1 | HLA-DQB1-AS1 | 0.482997 | 1.88E-06 | postive |
| IL18 | HLA-DQB1-AS1 | 0.537981 | 6.46E-08 | postive |
| NLRC4 | HLA-DQB1-AS1 | 0.482564 | 1.92E-06 | postive |
| NLRP3 | HLA-DQB1-AS1 | 0.572003 | 5.83E-09 | postive |
| PYCARD | HLA-DQB1-AS1 | 0.498855 | 7.55E-07 | postive |
| AIM2 | AP001434.1 | 0.443658 | 1.50E-05 | postive |
| GSDMB | AL162385.2 | 0.47171 | 3.50E-06 | postive |
| GSDMB | AC005726.3 | 0.470319 | 3.77E-06 | postive |
| IL6 | AL138921.2 | 0.513534 | 3.11E-07 | postive |
| AIM2 | AP001189.3 | 0.401314 | 0.000107 | postive |
| GSDME | AP001189.3 | 0.406859 | 8.36E-05 | postive |
| AIM2 | AC144831.1 | 0.434649 | 2.32E-05 | postive |
| PLCG1 | AL365330.1 | 0.412036 | 6.64E-05 | postive |
| CASP3 | AC024257.4 | -0.41303 | 6.35E-05 | negative |
| IL6 | AC024257.4 | 0.640151 | 1.88E-11 | postive |
| CASP1 | AC083862.2 | 0.402618 | 0.000101 | postive |
| IL6 | AC021087.3 | 0.452838 | 9.43E-06 | postive |
| IL6 | TPRG1-AS1 | 0.41463 | 5.91E-05 | postive |
| CASP3 | AGBL1-AS1 | -0.40358 | 9.66E-05 | negative |
| IL6 | AGBL1-AS1 | 0.640185 | 1.87E-11 | postive |
| CASP1 | AC090559.1 | 0.496956 | 8.44E-07 | postive |
| IL18 | AC090559.1 | 0.579334 | 3.35E-09 | postive |
| NLRC4 | AC090559.1 | 0.473854 | 3.11E-06 | postive |
| NLRP3 | AC090559.1 | 0.652546 | 5.64E-12 | postive |
| PYCARD | AC090559.1 | 0.538624 | 6.19E-08 | postive |
| GSDMC | AC110285.2 | 0.485179 | 1.66E-06 | postive |
| AIM2 | LINC01605 | 0.689472 | 1.10E-13 | postive |
| GSDME | LINC01605 | 0.501024 | 6.64E-07 | postive |
| PJVK | AC009159.3 | 0.486909 | 1.51E-06 | postive |
| GSDMD | C22orf34 | 0.413682 | 6.17E-05 | postive |
| IL18 | C22orf34 | 0.409976 | 7.29E-05 | postive |
| PYCARD | C22orf34 | 0.566783 | 8.59E-09 | postive |
| NLRP1 | AF117829.1 | 0.420398 | 4.54E-05 | postive |
| ELANE | Z69706.1 | 0.504897 | 5.27E-07 | postive |
| CASP1 | CARD8-AS1 | 0.574531 | 4.82E-09 | postive |
| CASP8 | CARD8-AS1 | 0.438451 | 1.93E-05 | postive |
| IL18 | CARD8-AS1 | 0.601247 | 5.86E-10 | postive |
| NLRC4 | CARD8-AS1 | 0.441242 | 1.68E-05 | postive |
| NLRP3 | CARD8-AS1 | 0.5877 | 1.75E-09 | postive |
| PYCARD | CARD8-AS1 | 0.511198 | 3.59E-07 | postive |
| SCAF11 | AL355075.2 | 0.416561 | 5.42E-05 | postive |
| NLRC4 | AP003392.1 | -0.40247 | 0.000101 | negative |
| PYCARD | AP003392.1 | -0.44938 | 1.12E-05 | negative |
| IL18 | AL583785.1 | 0.407106 | 8.27E-05 | postive |
| NLRC4 | AL583785.1 | 0.446434 | 1.30E-05 | postive |
| PYCARD | AL583785.1 | 0.407866 | 8.00E-05 | postive |
| GSDMC | AL162171.2 | 0.58092 | 2.96E-09 | postive |
| PJVK | TTC28-AS1 | 0.447871 | 1.21E-05 | postive |
| GSDMB | AC124312.3 | 0.407673 | 8.07E-05 | postive |
| NLRP1 | LINC01278 | 0.43981 | 1.81E-05 | postive |
| GSDMC | HSD11B1-AS1 | 0.571756 | 5.94E-09 | postive |
| GSDMC | ACAP2-IT1 | 0.639207 | 2.05E-11 | postive |
| IL6 | AL121992.2 | 0.678752 | 3.67E-13 | postive |
| IL6 | AC100847.1 | 0.443666 | 1.49E-05 | postive |
| CASP4 | AC017033.1 | 0.416798 | 5.36E-05 | postive |
| NLRP1 | ALOX12-AS1 | 0.55179 | 2.51E-08 | postive |
| NLRP1 | ZFPM2-AS1 | 0.432452 | 2.58E-05 | postive |
| GSDMB | CCDC18-AS1 | 0.490407 | 1.23E-06 | postive |
