# Supplementary figures and images for: Prognostic Value of a Pyroptosis-Related Long Noncoding RNA Signature Associated with Osteosarcoma Microenvironment
Source: J Oncol. 2021 Nov 11;2021:2182761. doi: 10.1155/2021/2182761 (PMC8601829; doi:10.1155/2021/2182761)

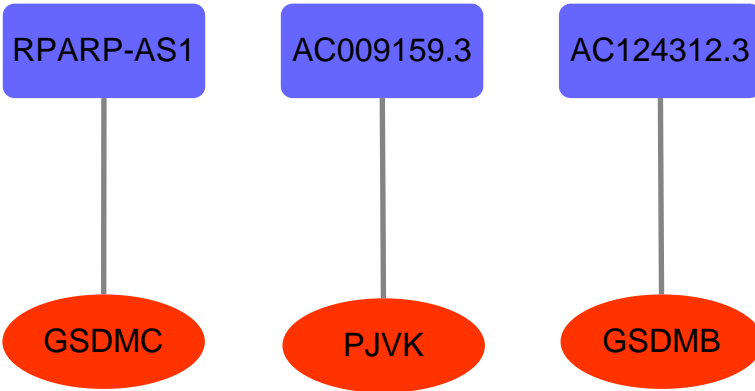

Supplement: Supplementary Materials — Supplementary File Table S1. 33 pyroptosis-related genes from prior reviews. Supplementary File Table S2. Patients' clinical features from the TARGET dataset. Supplementary File Table S3. Differential expression pyroptosis-related genes. Supplementary File Table S4. 329 pyroptosis-related lncRNAs by performing Pearson correlation analysis. Supplementary File Figure S1. The relationship between the novel lncRNA and mRNA. [file 2182761.f1.zip › 2182761.f1/FigureS1.pdf]
